# Machine learning analysis of complex late gadolinium enhancement patterns to improve risk prediction of major arrhythmic events

**DOI:** 10.3389/fcvm.2023.1082778

**Published:** 2023-02-07

**Authors:** Hassan A. Zaidi, Richard E. Jones, Daniel J. Hammersley, Suzan Hatipoglu, Gabriel Balaban, Lukas Mach, Brian P. Halliday, Pablo Lamata, Sanjay K. Prasad, Martin J. Bishop

**Affiliations:** ^1^Department of Biomedical Engineering, School of Biomedical and Imaging Sciences, King’s College London, London, United Kingdom; ^2^National Heart and Lung Institute, Imperial College London, London, United Kingdom; ^3^Cardiovascular Magnetic Resonance Unit, Royal Brompton and Harefield Hospitals, Guy’s and St Thomas’ NHS Foundation Trust, London, United Kingdom; ^4^Department of Computational Physiology, Simula Research Laboratory, Oslo, Norway

**Keywords:** late gadolinium enhanced, cardiovascular magnetic resonance, sudden cardiac death, coronary artery disease, arrhythmic risk stratification, scar heterogeneity, fibrosis, entropy

## Abstract

**Background:**

Machine learning analysis of complex myocardial scar patterns affords the potential to enhance risk prediction of life-threatening arrhythmia in stable coronary artery disease (CAD).

**Objective:**

To assess the utility of computational image analysis, alongside a machine learning (ML) approach, to identify scar microstructure features on late gadolinium enhancement cardiovascular magnetic resonance (LGE-CMR) that predict major arrhythmic events in patients with CAD.

**Methods:**

Patients with stable CAD were prospectively recruited into a CMR registry. Shape-based scar microstructure features characterizing heterogeneous (‘peri-infarct’) and homogeneous (‘core’) fibrosis were extracted. An ensemble of machine learning approaches were used for risk stratification, in addition to conventional analysis using Cox modeling.

**Results:**

Of 397 patients (mean LVEF 45.4 ± 16.0) followed for a median of 6 years, 55 patients (14%) experienced a major arrhythmic event. When applied within an ML model for binary classification, peri-infarct zone (PIZ) entropy, peri-infarct components and core interface area outperformed a model representative of the current standard of care (LVEF<35% and NYHA>Class I): AUROC (95%CI) 0.81 (0.81–0.82) vs. 0.64 (0.63–0.65), *p* = 0.002. In multivariate cox regression analysis, these features again remained significant after adjusting for LVEF<35% and NYHA>Class I: PIZ entropy hazard ratio (HR) 1.88, 95% confidence interval (CI) 1.38–2.56, *p* < 0.001; number of PIZ components HR 1.34, 95% CI 1.08–1.67, *p* = 0.009; core interface area HR 1.6, 95% CI 1.29–1.99, *p* = <0.001.

**Conclusion:**

Machine learning models using LGE-CMR scar microstructure improved arrhythmic risk stratification as compared to guideline-based clinical parameters; highlighting a potential novel approach to identifying candidates for implantable cardioverter defibrillators in stable CAD.

## 1. Introduction

Despite well-documented limitations, existing clinical strategies to determine sudden cardiac death (SCD) risk and guide implantable cardioverter defibrillator candidacy remain centered on left ventricular ejection fraction (LVEF) (1). This approach is insensitive and non-specific with the majority of SCD occurring in patients not incorporated in current clinical guidelines and only a small proportion of the patients who undergo device implantation receiving an appropriate shock (2, 3). Conversely, there is increasing data supporting the utility of myocardial scar quantification by LGE-CMR to predict SCD (4, 5). This body of work is importantly underpinned by the plausible causal relationship between myocardial fibrosis and ventricular arrhythmia. Furthermore, there is growing research detailing the prognostic role of shape-based scar microstructure features. Our group recently published (6–9) the role of morphological and texture-related scar features in both ischaemic heart disease (4) and dilated cardiomyopathy (6–9). The characterization of these features is, however, complex with multiple potential methods to quantify and aggregate the signal from the core scar and peri-infarct zone (PIZ). To circumvent these issues, computational analysis techniques can be used to rapidly assess multiple methodologies of LGE characterization. Additionally, there exist numerous limitations in the application of traditional regression analysis to inform changes in clinical practice. Conversely, machine learning approaches have the potential to explore nonlinear relationships and provide binary decisions. This has notable potential utility concerning guiding implantable cardioverter defibrillator recommendations.

Previous studies suggest that the heterogeneous texture of scar provides a significant pro-arrhythmogenic substrate (9–12). Specifically, prior research has shown LGE scar heterogeneity to be an independent risk predictor on a small cohort of ischemic cardiomyopathy (13), and that the peri-infarct region provides incremental prognostic value above clinical benchmarks (10, 12). In a non-ischemic cohort (8, 9), the LGE-myocardial interface area, along with features such as entropy and number of fibrotic components, were shown to be associated with arrhythmic risk. Finally, computer vision analysis through local binary patterns have shown early promise in a small cohort (14) of post-MI patients, despite being more challenging to interpret clinically. These findings suggest that a more granular examination of the heterogeneous (‘peri-infarct’) and homogeneous (‘core’) fibrosis, could uncover more robust image-based biomarkers, which when used within commonly adopted machine learning (ML) strategies, could enable the exploration of complex feature combinations leading to improved discrimination above traditional clinical modeling methods.

Our study aimed to assess the use of computational modeling of scar microstructure features, in combination with a machine learning analytical approach, to improve the prediction of major arrhythmic events (MAE). We seek to identify new scar microstructure insight than in previous works (9).

## 2. Methods

### 2.1. Patient recruitment

Patients referred to our center at the Royal Brompton & Harefield NHS Hospital trust for late gadolinium enhancement cardiovascular magnetic resonance (LGE-CMR) evaluation of ischaemic heart disease were recruited between 2009 and 2016. All patients provided written consent. CMR was performed on a 1.5 Tesla scanner (Sonata/Avanto, Siemens). LGE images were acquired following intravenous injection of a gadolinium-based contrast agent (0.1 mmol/Kg). An inversion recovery gradient echo sequence was subsequently undertaken at 10 min, as described previously (15). The slice thickness was 8 mm with a 2 mm gap. Inversion times were optimized to ensure adequate nulling of normal myocardium (characteristically between 280 ms and 400 ms). LGE quantification of the core infarct and PIZ was performed by a Level 3 accredited CMR operator (SH) blinded to the clinical outcomes. The LGE slices were then separately reviewed by a second accredited CMR operator (RJ).

The inclusion criteria for the study were significant epicardial CAD (≥75% stenosis in the left main stem/proximal left anterior descending artery or ≥ 75% in 2 other epicardial coronary arteries), prior coronary revascularization, or clinical history of prior myocardial infarction (confirmed on CMR). Exclusion criteria were a class I indication for a secondary prevention ICD, myocardial infarction (MI) within 40 days prior to CMR, severe organic valve disease, absence of LGE, previous valvular intervention, or primary dilated, hypertrophic or infiltrative cardiomyopathy.

Of 734 eligible patients, 257 were excluded on diagnosis, 9 for technical reasons, 31 lost to follow-up and 40 to no scar. This allowed 397 high quality LGE-CMR patients with complete follow-up for our study.

### 2.2. Late gadolinium enhancement quantification

Epicardial and endocardial contours were drawn using CVI42 (Circle Cardiovascular Imaging Inc., Calgary, Canada) from the short-axis LGE slices. Expert CMR level 3 readers marked core infarct, peri-infarct zone (PIZ) and remote regions. Quantification was by Full Width Half Maximum (FWHM) and Standard Deviation (SD) methodologies. A 10% endocardial and epicardial erosion was used. In the FWHM analysis, core infarct was defined as any region with a signal intensity (SI) of >50% of maximal SI and the peri-infarct zone (PIZ) a 35–50% of the maximum SI. For the standard deviation methodology, 2 approaches were used:

- (2,3 SD), core infarct was defined as >3SD of the mean remote myocardium SI and PIZ as 2-3SD of the mean remote myocardium SI.
- (2,5 SD), core infarct was defined as >5SD of the mean remote myocardium SI and PIZ as 2-5SD of the mean remote myocardium SI.

### 2.3. 2-Dimensional scar microstructure features

**Figure 1:**
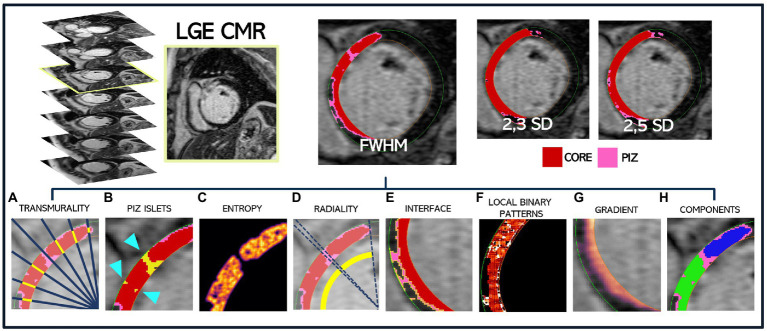
Extraction methodology for a single patient LGE-CMR DICOM slice to obtain the corresponding FWHM, 2,3SD and 2,5SD quantification masks, where core scar is shown in red and PIZ in pink. All scar features are then extracted and shown for the FWHM mask only. **(A)** Core transmurality by ray tracing (a small subset of rays are shown) **(B)** PIZ islets, shown by the blue arrow, surrounded by red core infarct **(C)** core entropy **(D)** core radiality **(E)** PIZ interface length shown in yellow, bounded by core scar and healthy myocardium **(F)** local binary pattern for total scar **(G)** gradient of total scar **(H)** core components, with blue and green regions depicting two separate areas of core scar.

We used LGE-CMR images and corresponding quantification masks to extract 2D morphological and texture-related scar features (Figure 1). All values (except PIZ islets) were calculated for the core scar, PIZ and the combined core scar and PIZ segmented regions (Table 1). Graphic descriptions of these metrics are shown in Figure 1 for an example patient.

**Table 1:** 2D LGE-CMR microstructure feature descriptions.

Feature name	Description
Entropy (9, 13, 16)	The level of disorder within the LGE. Calculated by applying standard Shannon entropy.
Transmurality (9, 17)	The extent of spread of LGE emanating outwards from the endocardium to epicardium, calculated using a ray tracing method.
Radiality (9)	Quantification of the angular spread of LGE in relation to the centre of the LV blood pool.
PIZ islets (18)	Regions of PIZ contained within core scar or an area of PIZ encapsulated by core scar and either endocardial or epicardial boundaries.
Number of components (9)	The degree of connectivity between LGE across the LV.
Interface area (9)	The extent of the border between myocardium and LGE.
Gradient (10, 14)	The rate of change in LGE intensity.
Local Binary Patterns (14)	Visual descriptor of texture with a selection of neighborhood radius sizes.

The scar features were computed for each slice (core infarct, PIZ and combined core + PIZ) and then aggregated across the short-axis stack to better detail the LGE throughout the LV. As an example, myocardium interface length (mm) is calculated for a slice and then multiplied by the slice thickness to calculate an interface area of the slice (mm^2^). This is then aggregated across the patient slices to obtain a single metric for the whole LV for an individual patient. This is repeated for each quantification method (FWHM; 2,3SD and 2,5SD).

### 2.4. Clinical endpoint

Patients were followed using health questionnaires in addition to primary and secondary care documentation. ICD reports, death certificates and post-mortem results were requested where necessary. Survival status was confirmed *via* the UK NHS Digital service to ensure that no deaths were missed. The duration of follow-up was determined from the date of CMR prior to consent until an endpoint was confirmed or until the most recent patient contact date. Event times were calculated from the date of the preceding CMR date for ≤10 years. All clinical outcomes were adjudicated by an independent panel of cardiologists blinded to the LGE data.

The endpoint was a composite of major arrhythmic events (MAE). This comprised SCD, aborted SCD or hemodynamically unstable VT. The time-to-event was taken as the earliest from any of the parameters. SCD was defined as a death that occurred unexpectedly, including scenarios where symptom duration was ≤1 h, following an identified arrhythmia/unsuccessful resuscitation or in circumstances where the patient was witnessed alive ≤24 h prior to death and without another identifiable cause of death (20). Aborted SCD was defined as appropriate ICD shock for a ventricular tachyarrhythmia, effective resuscitation following ventricular fibrillation or hemodynamically unstable VT requiring electrical cardioversion (19, 20).

### 2.5. Statistical methods

For baseline characteristics in the total cohort, frequency (%) was used for categorical variables compared with Fisher exact tests, and mean standard deviation (SD) compared with Mann–Whitney test for continuous variables. We performed time-to-event analysis and a separate binary classification discrimination. As all patients in the cohort had fibrosis on CMR, fibrosis presence was not considered as a variable for inclusion in the multivariable models. Analysis was performed on Python v3.7.4 and a *p*-value ≤ 0.05 was taken as significant.

For time-to-event analysis, we applied a univariate cox proportional hazard regression method to examine the association between the endpoint and various LGE quantification and microstructure aggregation techniques. Results are presented as hazard ratios (HRs) with 95% confidence intervals (CIs). Multivariate cox proportional hazard regression was also performed using covariates currently used (1) to risk stratify patients for ICD implantation; New York Heart Association (NYHA) functional class>1 and LVEF<35%. In additional analysis, we also investigated the importance of incorporating functional ECG covariates into the clinical benchmark. We assessed model performance using Harrel’s C-statistic.

Currently, an ICD recommendation is a binary decision usually made clinically from the intersection of LVEF (<35%) and NYHA (>Class I). Thus, a binary classifier that outperforms LVEF (<35%) and NYHA (>Class I) represents a promising investigation with real value. Feature class means were compared using Mann–Whitney tests.

We used the selection of ML classification methods to ensure the features selected are classifier independent: 5-Nearest Neighbors vote, Polynomial Support Vector Machine, Gaussian Process, Random Forest, Multi-layer Perceptron, Gaussian Naïve Bayes, Logistic Regression, Extra Trees ensemble and Linear Discriminant Analysis. In addition, given the importance of time-to-event modeling, we separately investigated the survival forests method across the feature space.

We sampled with a shuffled 10-fold cross-validation, evaluating performance using the Area Under the Receiver Characteristic Curve (AUROC). We consider differences between AUROC for MAE as significantly higher if 95% confidence intervals (CI) do not overlap.

We determined outliers and applied a selection of class balancing methods and averaged results across them, including random oversampling with replacement, Synthetic Minority Oversampling Technique (SMOTE) (21) and Adaptive Synthetic Sampling Method (ADASYN) (22).

We used a collection of methods to obtain a subset of features that provide the best descriptors for the arrhythmic endpoint, including Extra Trees importance, Backward Elimination, Recursive Feature Elimination and Embedded Features. It is possible to use other measures such as AIC, or stepwise regression, however as we repeated our tests with variation, we satisfy ourselves that no new insight would be gained from any additional measures for feature ranking. Scar microstructure features aim to measure and explain the same region of interest in different ways, and as expected some of these measurements share a high degree of correlation with one another, especially where we investigated different methods of aggregating the 2D LGE-CMR scar features across the stack of the LV, i.e., by averaging or summing. While removing features with >0.9 correlation, we keep features which remain correlated but measure different aspects of the scar areas. For example, the LGE-myocardium interface area is correlated with PIZ level of disorder (entropy), but both features are inherently different in how they are calculated and what they present. Co-linearity between features was assessed by variance inflation factor (VIF) with a value between 1 and 5 acceptable. All final model variables were within this acceptable range.

## 3. Results

### 3.1. Population characteristics

A total of 397 patients were included (mean age 64.4 ± 9.8 years, 346 (87%) male, and mean LVEF 45.4 ± 16%). The baseline characteristics of the study cohort are shown in Table 2. The patients were followed up for a median (IQR) of 6 (3) years, during which 55 (14%) patients experienced MAE. The patients that experienced MAE were older (*p* = 0.026), had a higher body surface area (*p* = 0.028) and a higher proportion were prescribed aldosterone antagonists (*p* = 0.003), loop diuretics (*p* = 0.002) or amiodarone (*p* = 0.021).

**Table 2:** MAE baseline patient characteristics.

Baseline patient characteristics: major arrhythmic event				
	Total Cohort (*n* = 397)	No Event (*n* = 342)	Event (*n* = 55)	*p*-value
Age, years	64.4 ± 9.8	64.8 ± 9.9	62.2 ± 8.9	0.026*
Male	346 (87.2%)	294 (86.0%)	52 (94.5%)	0.085
Body surface area (m^2^)	2.0 ± 0.2	1.95 ± 0.2	2.02 ± 0.2	0.028*
Heart rate (beats/min)	69.3 ± 13.2	69.1 ± 12.9	70.5 ± 14.9	0.581
Systolic blood pressure (mm Hg)	125.5 ± 18.5	125.9 ± 17.9	122.4 ± 21.6	0.147
Diastolic blood pressure (mm Hg)	72.9 ± 11.3	73.1 ± 11.0	71.3 ± 13.0	0.334
Diabetes	117 (29.5%)	100 (29.2%)	17 (30.9%)	0.874
Smoker (current)	41 (10.3%)	31 (9.1%)	10 (18.2%)	0.053
Hypertension	207 (52.1%)	179 (52.3%)	28 (50.9%)	0.885
Prior MI	302 (76.1%)	257 (75.1%)	45 (81.8%)	0.312
Family history of premature coronary artery disease	89 (22.4%)	77 (22.5%)	12 (21.8%)	1
**NYHA functional class**				
I	131.0 (33.0%)	117.0 (34.2%)	14.0 (25.5%)	0.22
II	183.0 (46.1%)	154.0 (45.0%)	29.0 (52.7%)	0.31
III/IV	81.0 (20.4%)	69.0 (20.2%)	12.0 (21.8%)	0.857
**Medications**				
Beta-blocker	311 (78.3%)	266 (77.8%)	45 (81.8%)	0.598
ACE inhibitor/ARB	345 (86.9%)	293 (85.7%)	52 (94.5%)	0.084
Aldosterone antagonist	105 (26.4%)	81 (23.7%)	24 (43.6%)	0.003*
Loop diuretic	195 (49.1%)	157 (45.9%)	38 (69.1%)	0.002*
Lipid-lowering	351 (88.4%)	299 (87.4%)	52 (94.5%)	0.172
Calcium channel blocker	76 (19.1%)	68 (19.9%)	8 (14.5%)	0.46
Amiodarone	22 (5.5%)	15 (4.4%)	7 (12.7%)	0.021*
**CMR measurements**				
LV ejection fraction (%)	45.4 ± 16.0	46.7 ± 16.0	37.1 ± 13.2	<0.001*
LV end diastolic volume index (ml/m^2^)	109.9 ± 40.8	106.2 ± 38.1	132.9 ± 48.3	<0.001*
LV mass indexed (g/m^2^)	80.3 ± 25.1	78.7 ± 24.4	89.8 ± 26.7	0.002*
RV ejection fraction (%)	57.6 ± 12.9	58.1 ± 12.6	54.7 ± 14.4	0.059
**ECG measurements**				
Ventricular rate	70.7 ± 15.0	70.3 ± 14.4	73.0 ± 18.1	0.443
PR interval (ms)	171.4 ± 32.1	170.6 ± 31.3	176.3 ± 35.8	0.264
QRS duration (ms)	106.4 ± 23.2	105.7 ± 23.3	110.9 ± 21.7	0.032*
QTc interval (ms)	436.6 ± 35.4	435.0 ± 35.0	446.9 ± 35.7	0.016*

Mean ± SD, compared with Mann–Whitney tests, or *n* (%), compared with Fisher exact tests. ^*^Statistically significant (*p* < 0.05). ACE, angiotensin-converting enzyme; ARB, angiotensin receptor blocker; CMR, cardiac magnetic imaging; LV, left ventricular; RV, right ventricular; NYHA, New York Heart Association.

The mean (SD) LVEF for patients who experienced MAE was lower than for those that did not [37.1% (13.2) vs. 46.7% (16.0)], yet there was no difference in NYHA class. Over the course of follow-up, 108 (27%) patients received an ICD, 105 (26%) of whom also received cardiac resynchronization therapy.

### 3.2. Time-to-event cox proportional hazards

**Figure 2:**
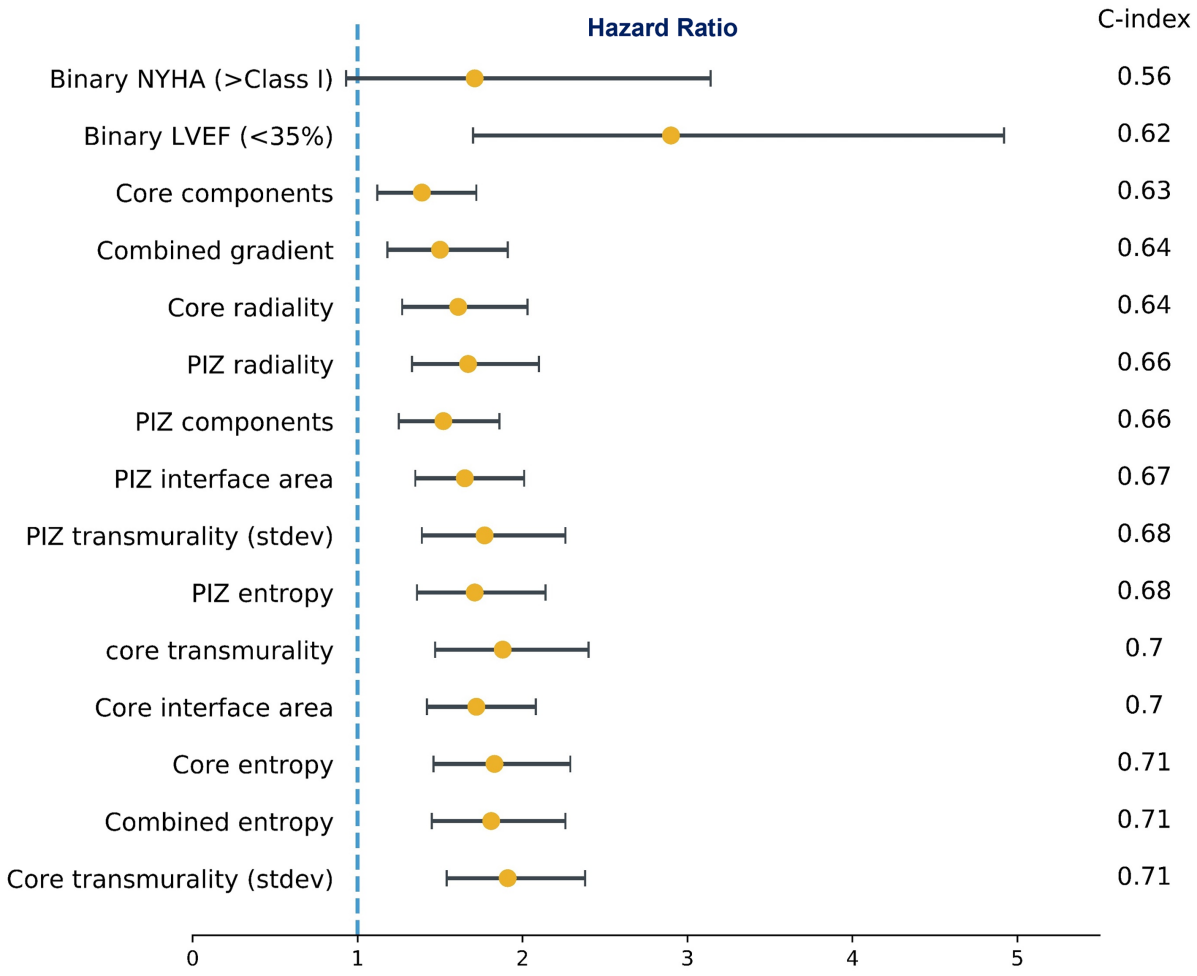
Subset of univariate cox proportional hazards regression models for microstructure features and the clinical benchmark. Hazard Ratios (95% CI) and C-index. NYHA (>Class I) was not significant (*p* = 0.08), while all other features were significant at *p* ≤ 0.05. The values shown are for FWHM only.

Univariate cox proportional hazard models for the scar microstructures demonstrated a substantial number of features which were associated with the primary endpoint irrespective of the FWHM, 2,3SD and 2,5SD quantification methods used. 2D scar microstructure features were retained if the C-statistic was above the clinical benchmark (LVEF <35% and NYHA >1) and the feature remained significant (*p* ≤ 0.05). The best-performing features are presented in Figure 2. Overall, we identified that aggregating the features using the sum of the feature value resulted in higher model discrimination when compared to using the mean in the majority of scar features.

**Figure 3:**
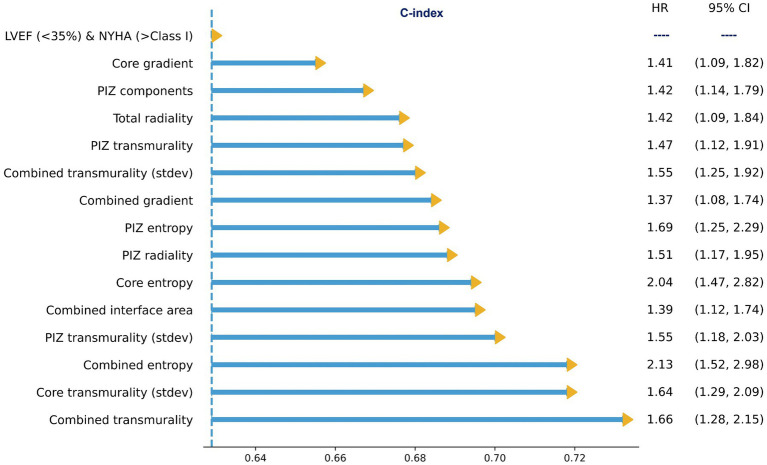
Subset of multivariate cox proportional hazard regression models with the clinical benchmark, LVEF<35% and NYHA >Class I, as covariates. All microstructure features remained significant (*p* ≤ 0.05). The values shown are for FWHM only.

On multivariate cox regression analysis, we iterated across all features that were significant in the univariate cox models, and the features presented in Figure 3 remained significant (*p* ≤ 0.05), improved performance over the clinical benchmark LVEF (<35%) and NYHA (>Class I) C-index 0.629, and had the lowest correlation among the other features. These features were independent of the LGE quantification method. Therefore, following from the results in Figures 2, 3, we identify a subset of viable microstructure features which improve upon the clinical benchmark and could hold utility in a binary predictive model for MAE.

### 3.3. Binary major arrhythmic events classification

We performed Mann–Whitney tests on feature mean differences, identifying a large selection of features that had a significant (*p* ≤ 0.05) difference in group means and had a higher AUROC than the clinical benchmarks. The best-performing features are presented in Table 3.

**Table 3:** Subset of the univariate analysis of scar microstructure for core and PIZ scar, shown for FWHM values.

Univariate significant microstructure features: major arrhythmic event			
Feature across whole LV	No event mean (*n* = 342)	Event mean (*n* = 55)	AUROC
Combined transmurality	4.22 ± 1.91	5.7 ± 1.61	0.73 ± 0.14
Core entropy	23.29 ± 8.96	29.61 ± 6.63	0.73 ± 0.12
Core transmurality	3.43 ± 1.66	4.69 ± 1.5	0.72 ± 0.13
Core interface area	9420.09 ± 5,840	14475.93 ± 6792.99	0.72 ± 0.14
PIZ transmurality (standard deviation)	1.19 ± 0.52	1.53 ± 0.44	0.7 ± 0.14
PIZ entropy	16.44 ± 6.77	20.53 ± 5.09	0.7 ± 0.13
Combined interface area	11114.46 ± 6806.83	15909.75 ± 7769.66	0.68 ± 0.1
PIZ interface area	14451.89 ± 9423.88	21241.53 ± 11535.51	0.67 ± 0.1
PIZ components	134.08 ± 80.4	184.09 ± 88.39	0.67 ± 0.14
PIZ radiality	2.61 ± 1.62	3.68 ± 1.82	0.66 ± 0.15
Core radiality	1.86 ± 1.35	2.65 ± 1.51	0.65 ± 0.17
Combined gradient	5.74 ± 2.08	6.77 ± 1.7	0.65 ± 0.11
Core gradient	5.42 ± 2.02	6.25 ± 1.56	0.64 ± 0.11
Binary LVEF (<35%)			0.61 ± 0.13
Binary NYHA (>Class I)			0.55 ± 0.12

All features have a significant difference in no event vs. event means (*p* ≤ 0.05), irrespective of the quantification method (FWHM; 2,3SD; 2,5SD). Mean ± SD, all significant (*p* < 0.05) when compared with Mann–Whitney tests. LVEF, left ventricular ejection fraction; NYHA, New York Heart Association.

### 3.4. Multivariate machine learning ensemble

We applied the feature selection algorithms to obtain an independent subset of features, which consisted of features similar to those identified as being top performers in the survival Cox analysis in Figures 2, 3. Each feature selection algorithm provided an indication of the number of features and the best features to combine. Through an iterative search process, we identified the best average discrimination (no further significant improvement in 95% CI) to be with models with 3 features. In some combinations tested, we used multiple correlated features (e.g., core interface, PIZ interface and combined interface area) to examine whether there was any beneficial reduction in measurement errors *via* averaging.

Finally, given the importance of time-to-event modeling, we separately investigated survival forests across the feature space, but did not find any new insight beyond the significant findings already reported within the multivariate cox regression and the binary classification models (see Supplementary Table S2).

### 3.5. Machine learning identified scar microstructure features

We iterated across a varying number of parameters and model sizes, with the best-performing ML classifier features shown in Table 4.

**Table 4:** Subset of top-performing core and PIZ features using a selection of ML models.

ML ensemble: major arrhythmic event		
Feature names	Features (*n*)	Mean AUROC (95% CI)
Core transmurality, Combined interface area, Combined entropy, Core interface area, Core gradient, Core radiality, LVEF (<35%), NYHA (>Class I)	8	0.815 (0.808,0.8222)
Core transmurality (standard deviation), Core radiality, Combined interface area, PIZ interface area, Combined transmurality, Combined entropy, Core gradient	7	0.815 (0.807,0.823)
Core transmurality (standard deviation), Core radiality, Combined entropy, Core interface area, Combined interface area	5	0.814 (0.807,0.821)
PIZ entropy, PIZ components, Core interface area	3	0.812 (0.806,0.819)^*^
Combined interface area, Combined transmurality, Combined entropy, Core interface area	4	0.811 (0.799,0.823)
Core transmurality (standard deviation), Combined entropy, PIZ interface area, Core interface area, Combined interface area, Core gradient	6	0.811 (0.806,0.817)
PIZ components, PIZ entropy	2	0.765 (0.755,0.776)
LVEF (<35%) and NYHA (>Class I)	2	0.643 (0.633,0.653)

The values shown are for FWHM. Mean AUROC across shuffled 10-fold cross-validation. AUROC are significantly higher than LVEF (<35%) and NYHA (>Class I) baseline (95% confidence intervals do not overlap). ^*^Indicates the best performance for the number of features.

The best performing scar microstructure features were found to be PIZ entropy, number of PIZ components and core interface area. The simplest model (least parameters) which outperformed the clinical benchmark model comprised of the number of PIZ components and PIZ entropy; the addition of the core interface area provided the best discrimination for the least number of parameters, where after subsequent additional parameters were unable to significantly improve the performance (95% CI overlap).

We found that the addition of the core interface area to PIZ entropy and PIZ components was not unique and that similar improvements (no significant *p* ≤ 0.05 difference between AUROCs) could be made by replacing the core interface area with another microstructure (Table 5). Therefore, we interpret this as the PIZ entropy and PIZ components being the driving features for the majority of the improvement above the clinical parameters for MAE classification.

**Table 5:** Non-significant differences in AUROC for the additional parameter for PIZ entropy and PIZ components.

ML 3 parameters: major arrhythmic event		
Feature names	Features (*n*)	Mean AUROC (95% CI)
PIZ entropy, PIZ components, Core transmurality (standard deviation)	3	0.812 (0.806,0.819)
PIZ entropy, PIZ components, Core interface area	3	0.801 (0.791,0.811)
PIZ entropy, PIZ components, Combined entropy	3	0.794 (0.789,0.802)

The values shown are for FWHM. Mean AUROC across shuffled 10-fold cross-validation. AUROC differences are not significant (95% confidence intervals overlap).

### 3.6. Risk in high and low left ventricular ejection fraction patients

In addition, we also considered the performance of the models in the sub-groups of patients with both preserved and severely impaired systolic function. In the cohort of preserved LVEF above 50%, 159 patients with 11 (7%) had a MAE. The clinical benchmark of LVEF and NYHA was outperformed (C-index 0.6 to 0.8) by PIZ entropy (*p* = 0.001) (See Supplementary Table S3). In the severe systolic dysfunction sub-group LVEF <35%, 28 (24%) of 115 patients had a MAE, and the number of PIZ components (*p* = 0.014) outperformed (C-index 0.54 to 0.66) the clinical benchmark (See Supplementary Table S4). Note that, although instructive, the smaller populations in this sub-group analysis, combined with the relatively low overall event-rate in our population as a whole (compared to, for example, an ICD-cohort), meant that we did not have sufficient statistical power (0.8) to draw robust conclusions in these sub-groups.

## 4. Discussion

There is increasing data detailing the use of complex myocardial scar modeling to identify patients at increased risk of life-threatening ventricular arrhythmia. In this study, we demonstrate the use of machine learning to identify scar microstructures that are significantly associated with arrhythmic risk. Numerous features were independent of the scar quantification method used and stratified patients beyond the current clinical benchmark of LVEF <35% and NYHA >1 with two features of particular interest: Number of PIZ components and PIZ entropy.

### 4.1. Machine learning approach

The features identified through the binary classification ML models corroborate with the features identified in the time-to-event Cox regression results, demonstrating the model-agnostic high discriminative power for PIZ entropy and the number of PIZ components. Importantly these features are independent of the LGE quantification method, remaining significant using FWHM, 2,3SD and 2,5SD techniques. This is important as there can be a considerable difference in the quantity of LGE that is identified depending on the type of signal intensity quantification method used.

### 4.2. Measuring disorder within the peri-infarct zone: Entropy

**Figure 4:**
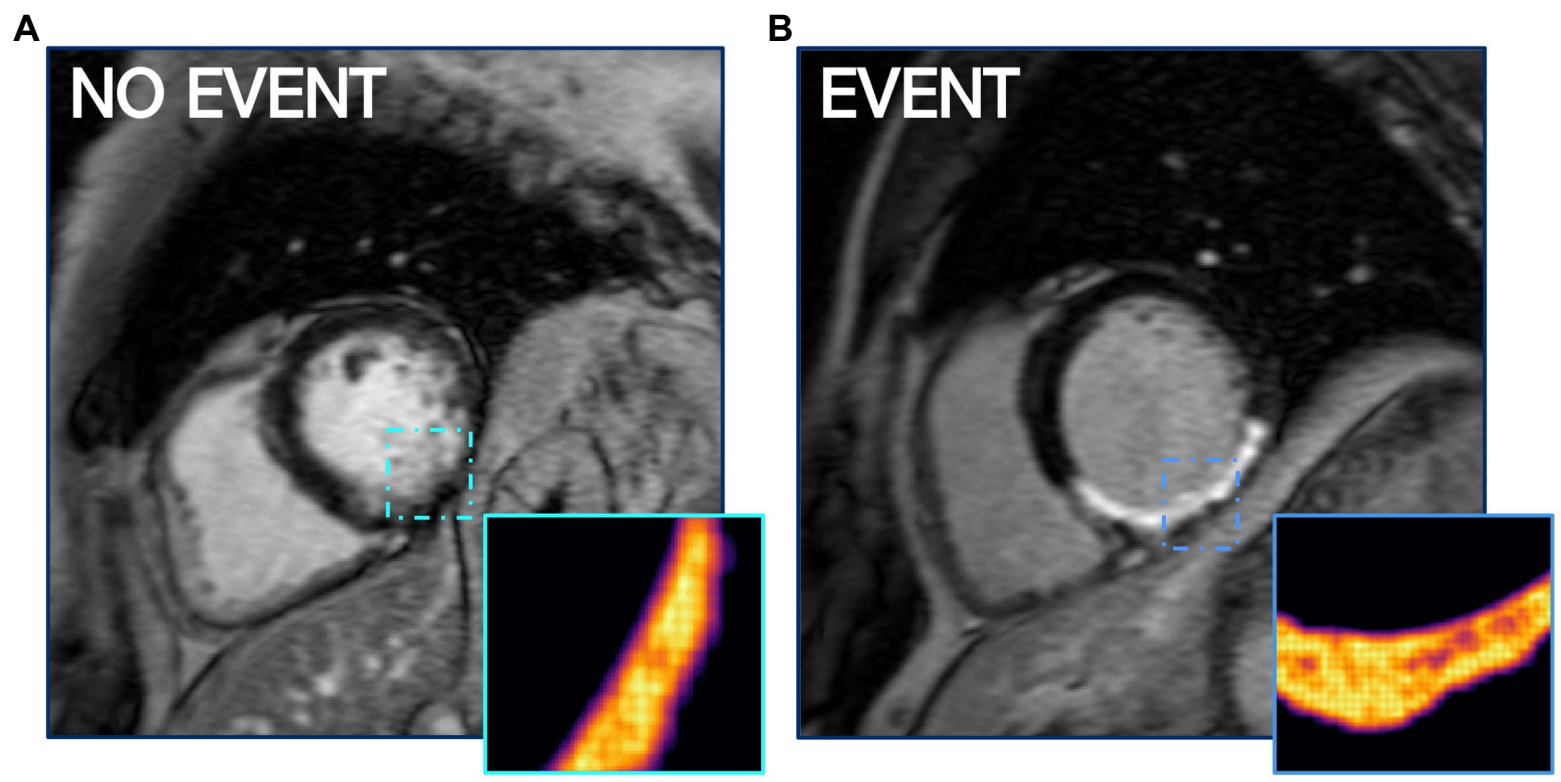
PIZ Entropy is significantly (*p* ≤ 0.05) higher in patients who have MAE, as seen in **(B)** compared to a non-event patient LGE CMR shown in **(A)**. Images depict entropy *via* FWHM quantification.

Entropy describes the level of disorder within the region of a scar. This metric was included in all 4 best performing models as either PIZ entropy or combined entropy (Table 4), and suggests that patients with higher arrhythmic risk have a more complicated composition of viable myocytes and fibrotic components in the PIZ region (Figure 4). The strength of PIZ entropy is in identifying subtle tissue characteristics by filtering out image noise and accentuating key features. Entropy has previously been shown to associate with ventricular arrhythmia (13, 16) and is potentially more reproducible than other measures of tissue characterization including T1 mapping (13).

Local binary patterns (LBP) may be considered another measure of disorder and have been reported as good discriminators of arrhythmic risk (14). While in our analysis we note that such features remain significant on multivariate analysis, they do not provide incremental information compared to other, more readily interpretable and easier to compute, scar features.

### 4.3. Characterising structural heterogeneity: Number of peri-infarct zone components

**Figure 5:**
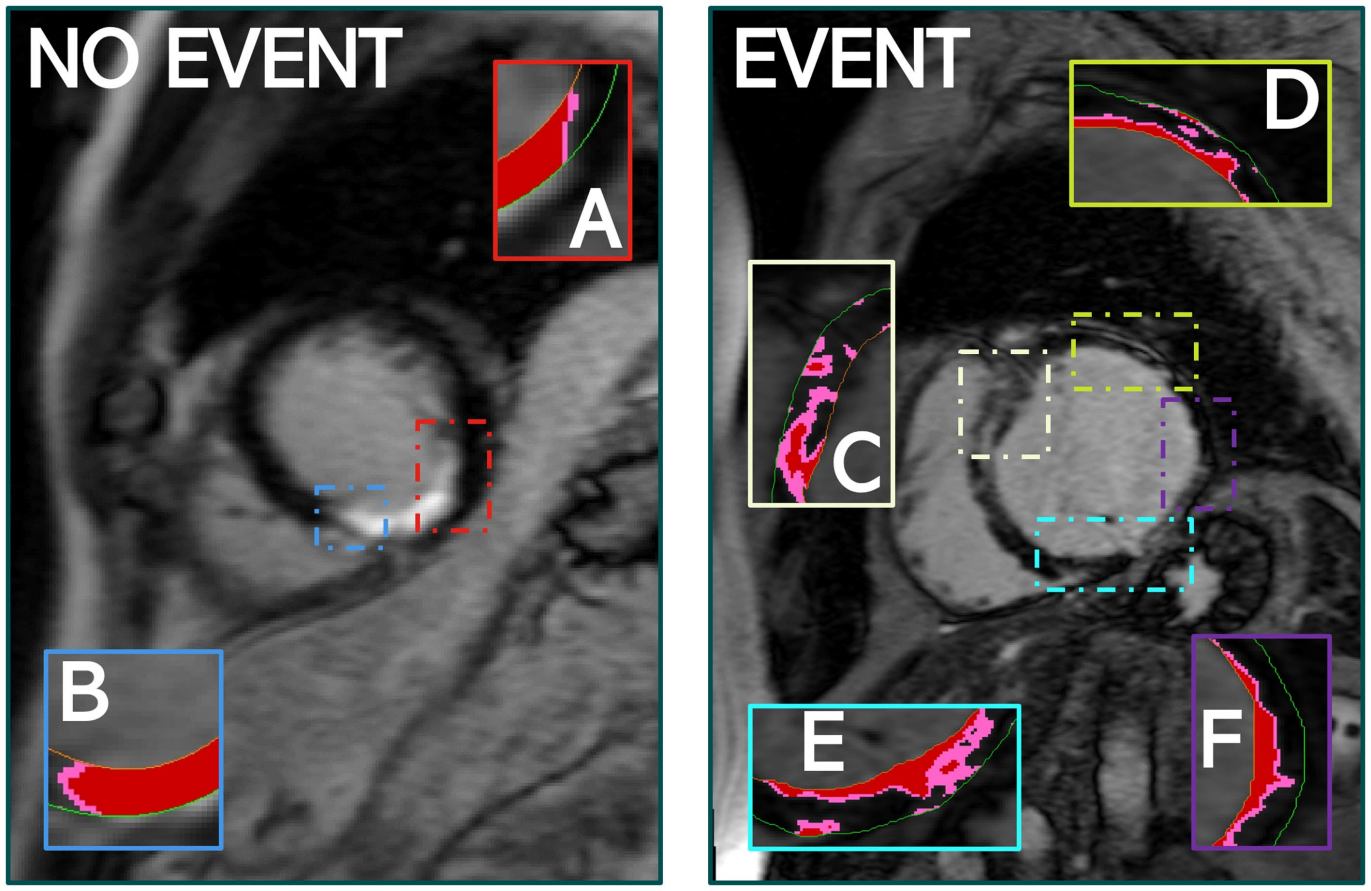
The quantity of PIZ components was significantly higher in patients that experienced MAE compared to those that did not, as seen above. **(A,B)** Identify PIZ components, shown in pink with core infarct in red, in a patient who did not experience MAE. **(C–F)** Show the variety and additional PIZ components in a patient who experienced MAE. Images used FWHM quantification.

The number of PIZ components details the unique number of PIZ elements across the LV. Our ML analysis consistently demonstrated that the number of PIZ components had important prognostic value in predicting MAE (Figure 5). This result continues to build on the hypothesis that these regions contain the arrhythmogenic substrate needed to initiate and maintain ventricular arrhythmia (23–25).

The total core transmurality and the variance of core transmurality across the LV were the other two features shown to provide the most incremental improvement to the clinical benchmark of LVEF (<35%) and NYHA (>Class I), and remained significant (*p* ≤ 0.05) in the multivariate time-to-event Cox analysis. We suggest that the highly transmural scars are potentially indicative of more mature infarcts and that the variance in transmurality across the LV stack may be indicative of viable isthmuses which can sustain re-entry around the core infarct regions.

### 4.4. Limitations

We use a large cohort representing the typical presentation of UK patients to the Royal Brompton & Harefield hospitals. Our study is, however, a single center and would benefit from external validation using a separate patient population with suitable LGE-CMR quantification and adequate patient follow-up data. We are actively engaging with colleagues in other UK hospital trusts to obtain suitable datasets, however, this is a very substantial undertaking. Our research is currently in the early stages of development and additional work needs to be done to mature the technology ready for clinical adoption. Although automated segmentation tools are becoming increasingly available to identify epi/endo contours, the detailed analysis of the scar (which requires selection of exclusion regions, and regions of interest) still requires significant clinical input. If taken forward as clinical risk stratification tool, we believe that the (semi) manual analysis time required to accurately identify scar on patient CMR required as input for our methodology would represent minimal additional clinical input relative to the total clinical interaction time per patient. The right ventricular scar was not evaluated, which may contribute to the overall scar burden and arrhythmic substrate. Although electrical abnormalities, such as T-wave alternans, heart rate variability, as recorded by the ECG, have been linked with risk of MAE in CAD (26, 27), in our dataset we only had access to more simple ECG features (QRS width, QT interval). Our analysis showed that these ECG features did not provide any meaningful additional contribution (see Supplementary Table S1). Future work with prospective cohorts would be required to thoroughly assess the potential importance of previously suggested more specialized ECG features (26, 27).

## 5. Conclusion

LGE-CMR myocardial fibrosis microstructure features offer enhanced arrhythmic risk stratification than conventional (LVEF<35%, NYHA>Class I) predictors and are reassuringly robust to quantification method (FWHM, 2,3SD and 2,5SD). Only 2 PIZ microstructure features are required to significantly improve performance above clinical predictors; highlighting a simple model of biologically plausible features that that could be used to tailor risk stratification for major arrhythmic events and ICD recommendations.

## Data availability statement

The data analyzed in this study is subject to the following licenses/restrictions: Images are available in anonymised form upon request, but are not publicly available due to patient privacy concerns. Appropriate institutional data transfer agreements will be required. Requests should be made, along with an analysis proposal, *via* email to the Research & Development team at Royal Brompton and Harefield NHS Foundation Trust. Requests to access these datasets should be directed to https://www.rbht.nhs.uk/research/research-office.

## Ethics statement

The data registry complied with the Declaration of Helsinki and the National Research Ethics Service approved the protocol. All patients provided informed written consent. The patients/participants provided their written informed consent to participate in this study.

## Author contributions

HZ and RJ were involved in preparing manuscripts, interpreting of results, and data analysis. HZ and GB developed an analytical framework for the study. RJ, DH, SH, and LM completed data acquisition and preparation of follow-up data. BH, PL, SP, and MB provided substantial contributions to the conception and design of the work. All authors contributed to manuscript revision, read, and approved the submitted version.

## Funding

This work was supported by the National Institute for Health Research Biomedical Research Center at Guy’s and St. Thomas’ Trust and King’s College, the Center of Excellence in Medical Engineering funded by the Wellcome Trust and Engineering and Physical Sciences Research Council (EPSRC; WT088641/Z/09/Z). MB is supported by a Medical Research Council New Investigator Grant (MR/N011007/1) and British Heart Foundation (Project grant PG/18/74/34077). This work was supported by EPSRC 2018/19 DTP-EP/R513064/1 grant. This work was supported by a National Heart and Lung Institute Foundation grant awarded to Professor Sanjay Prasad and Richard Jones. PL holds a Wellcome Trust Senior Research Fellowship (209450/Z/17/Z) and BH is funded by a BHF Intermediate Fellowship (FS/ICRF/21/26019).

## Conflict of interest

The authors declare that the research was conducted in the absence of any commercial or financial relationships that could be construed as a potential conflict of interest.

## Publisher’s note

All claims expressed in this article are solely those of the authors and do not necessarily represent those of their affiliated organizations, or those of the publisher, the editors and the reviewers. Any product that may be evaluated in this article, or claim that may be made by its manufacturer, is not guaranteed or endorsed by the publisher.

## Abbreviations

CAD: coronary artery disease
LGE: late gadolinium enhancement
CMR: cardiovascular magnetic resonance
ML: machine learning
LVEF: left ventricular ejection fraction
NYHA: New York Heart Association
CI: confidence interval
PIZ: Peri-infarct zone
SCD: sudden cardiac death
ICD: implantable cardioverter defibrillator
MAE: major arrhythmic events
VT: ventricular tachycardia
2D: 2-Dimensional
FWHM: full width half maximum
SD: standard deviation
LV: left ventricle
AUROC: area under the receiver operating characteristic
IQR: inter-quartile range
HR: Hazard ratio
