# Supplementary material for: Machine learning analysis of complex late gadolinium enhancement patterns to improve risk prediction of major arrhythmic events
Source: Front Cardiovasc Med. 2023 Feb 7;10:1082778. doi: 10.3389/fcvm.2023.1082778 (PMC9941157; doi:10.3389/fcvm.2023.1082778)
Supplement: Supplementary file 1 [file Data_Sheet_1.PDF]

Machine learning analysis of complex late gadolinium enhancement patterns to improve risk prediction of major arrhythmic events.

Supplementary Data

1. Cox Multivariate models with the inclusion of the ECG features.

In the table provided below, we see that the additional ECG measurements offer little to no improvement in C-index, especially when a 2D scar microstructure feature is added to the model. The additional ECG measurements increase the complexity of the model without any meaningful contribution.

Supplementary Table 1: Multivariate Cox Regression with ECG

Feature names	C-index	Feature Hazard Ratio (95% CI)	Feature p-Value	Features (n)
**LVEF<35% & NYHA>Class I**	0.629	----	----	2
+ combined transmurality	0.73	1.66 (1.28-2.15)	<0.001	3
+ combined entropy	0.72	2.13 (1.52-2.98)	<0.001	3
+ combined interface area	0.695	1.39 (1.12-1.74)	0.003	3
+ PIZ radiality	0.688	1.51 (1.17-1.95)	0.002	3
**LVEF<35%, NYHA>Class I & QRS duration**	0.659	----	----	3
+ combined transmurality	0.711	1.58 (1.21-2.06)	0.944	4
+ combined entropy	0.696	1.65 (1.28-2.13)	0.692	4
+ combined interface area	0.673	1.45 (1.16-1.82)	0.7	4
+ PIZ radiality	0.679	1.51 (1.16-1.95)	0.846	4
**LVEF<35%, NYHA>Class I, QRS duration & QTc interval**	0.65	----	----	4
+ combined transmurality	0.71	1.69 (1.29-2.2)	0.084	5
+ combined entropy	0.71	1.73 (1.34-2.23)	0.758	5
+ combined interface area	0.687	1.37 (1.09-1.72)	0.188	5
+ PIZ radiality	0.661	1.47 (1.14-1.9)	0.079	5

2. Evaluation of random survival forests for time-to-event risk stratification

The results collaborate with the binary classification results, namely that PIZ entropy, PIZ components and core interface area offer the best performance improvement while also being the least complex model (minimal number of model features).

Supplementary Table 2: Random Survival Forests

Feature names	Features (n)	C-index	Mean AUC (95% CI)
PIZ components, PIZ entropy	2	0.739	0.781 (0.757,0.805)
**LVEF<35% & NYHA>Class I**	2	0.564	0.604 (0.577,0.631)
+ Combined transmurality	3	0.731	0.712 (0.697,0.726)
+ Core transmurality (std dev)	3	0.674	0.629 (0.605,0.653)
+ Combined entropy	3	0.706	0.739 (0.714,0.764)
+ PIZ transmurality (std dev)	3	0.673	0.706 (0.684,0.728)
+ Combined interface area	3	0.68	0.739 (0.718,0.761)
+ Core entropy	3	0.704	0.719 (0.689,0.749)
**PIZ entropy, PIZ components, Core interface area**	**3**	**0.766**	**0.812 (0.792,0.832)***
LVEF<35%, NYHA>Class I, QRS duration & QTc interval	4	0.582	0.645 (0.614,0.675)
Combined interface area, Combined transmurality, Combined entropy, Core interface area	4	0.75	0.767 (0.757,0.778)
Core transmurality (std dev), Core radiality, Combined entropy, Core interface area, Combined interface area	5	0.773	0.786 (0.771,0.8)
Core transmurality (std dev), Combined entropy, PIZ interface area, Core interface area, Combined interface area, Core gradient	6	0.765	0.775 (0.76,0.79)
Core transmurality (std dev), Core radiality, Combined interface area, PIZ interface area, Combined transmurality, Combined entropy, Core gradient	7	0.751	0.747 (0.731,0.763)
Core transmurality, Combined interface area, Combined entropy, Core interface area, Core gradient, Core radiality, LVEF (<35%), NYHA (>Class I)	8	0.712	0.748 (0.732,0.763)

* indicates best performing AUROC than LVEF<35% & NYHA>Class I baseline (95% confidence intervals do not overlap).

3. Sub-group analysis of patients with preserved and impaired LV systolic function

We performed analysis on two patient groups derived from the original population, a cohort of preserved LVEF ≥50%, and the subset of only severe LV dysfunction LVEF <35%.

In the preserved LVEF above 50%, we have 159 patients with 11 (7%) having a major arrhythmic event. In the severe dysfunction below 35% we have 115 patients with 28 (24%) major arrhythmic events. In the preserved LVEF group, we note that the clinical benchmark model of LVEF and NYHA is outperformed (C-index 0.6 to 0.8) by PIZ entropy, with small improvement when additional features are included, which we believe to be influenced to our limited event rate. However, this analysis provides a promising signal that further research with additional validation cohorts is worth investigating. It is important to note that the clinical utility is that these features perform much better in the preserved LVEF cohort, and therefore is a better risk measure that current guidelines.

Supplementary Table 3: Cox Regression in subgroup with LVEF ≥ 50%

**Feature names**	**C-index**	**Feature 1 Hazard Ratio (95% CI)**	**Feature 2 Hazard Ratio (95% CI)**	**Feature 3 Hazard Ratio (95% CI)**	**Features (n)**
PIZ components (p=0.358)	0.60	1.28 (0.75 - 2.18)	----	----	1
PIZ entropy (p=0.001)	**0.80***	3.63 (1.73 - 7.61)	----	----	1
Core interface area (p=0.006)	0.76	1.97 (1.21 - 3.19)	----	----	1
PIZ components (p=0.09), PIZ entropy (p=0.01)	0.87	0.4 (0.15 - 1.08)	8.56 (2.85 - 25.73)	----	2
**LVEF %** (p=0.098) **& NYHA>Class I** (p=0.7)	0.60	0.93 (0.85 - 1.01)	0.78 (0.23 - 2.71)	----	2
+ combined transmurality (p=0.001)	0.87	1.02 (0.91 - 1.13)	1.07 (0.3 - 3.81)	3.52 (1.72 - 7.22)	3
+ combined entropy (p=0.002)	0.81	0.98 (0.89 - 1.09)	0.99 (0.28 - 3.52)	4.32 (1.72 - 10.85)	3
+ combined interface area (p=0.084)	0.69	0.93 (0.85 - 1.03)	0.65 (0.19 - 2.27)	1.64 (0.93 - 2.89)	3
PIZ components (p=0.074), PIZ entropy (p=0.012), Core interface area (0.588)	0.82	0.37 (0.1 - 1.35)	7.4 (1.98 - 27.72)	1.2 (0.45 - 3.23)	3

For the severe dysfunction group, we see the clinical benchmark model of LVEF and NYHA is outperformed, but not to the same degree as in the preserved cohort, (C-index 0.54 to 0.66). Additional features in the multivariate cox models did not outperform the univariate cox models, and where performance via C-index is the same in models, preference is given to the simpler (less features) models. In this group, it is the case that using PIZ components alone is a strong risk predictor over the clinical guidelines.

Supplementary Table 4: Cox Regression in subgroup with LVEF < 35%

**Feature names**	**C-index**	**Feature 1 Hazard Ratio (95% CI)**	**Feature 2 Hazard Ratio (95% CI)**	**Feature 3 Hazard Ratio (95% CI)**	**Features (n)**
PIZ components (p=0.014)	**0.66***	1.46 (1.08 - 1.97)	----	----	1
PIZ entropy (p=0.085)	0.58	1.33 (0.96 - 1.83)	----	----	1
Core interface area (p=0.005)	0.65	1.56 (1.14 - 2.12)	----	----	1
PIZ components (p=0.133), PIZ entropy (p=0.896)	0.70	1.39 (0.9 - 2.15)	1.03 (0.65 - 1.62)	----	2
**LVEF %** (p=0.281) **& NYHA>Class I** (p=0.42)	0.54	1.04 (0.97 - 1.12)	1.5 (0.56 - 4.06)	----	2
+ combined transmurality (p=0.006)	0.64	1.01 (0.95 - 1.09)	1.32 (0.48 - 3.61)	1.48 (1.12 - 1.96)	3
+ combined entropy (p=0.113)	0.61	1.01 (0.94 - 1.08)	1.37 (0.51 - 3.71)	1.31 (0.94 - 1.84)	3
+ combined interface area (p=0.034)	0.57	1.05 (0.98 - 1.13)	1.46 (0.53 - 3.99)	1.45 (1.03 - 2.03)	3
PIZ components (p=0.839), PIZ entropy (p= 0.731), Core interface area (p=0.167)	0.57	0.93 (0.46 - 1.88)	0.91 (0.55 - 1.53)	1.69 (0.8 - 3.58)	3
